# Neoadjuvant endocrine therapy for luminal breast cancer treatment: a first-choice alternative in times of crisis such as the COVID-19 pandemic

**DOI:** 10.3332/ecancer.2020.1027

**Published:** 2020-04-27

**Authors:** Covadonga Martí, José I Sánchez-Méndez

**Affiliations:** 1 Breast Cancer Unit, Hospital Universitario La Paz, 28046 Madrid, Spain; 2 Gynaecology Department, Hospital Universitario La Paz, 28046 Madrid, Spain

**Keywords:** breast cancer, luminal, endocrine therapy, neoadjuvant, COVID-19

## Abstract

The epidemiological emergency caused by CoV-2 (COVID-19) has changed priorities in breast cancer management. In those places where the pandemic has had the greatest effect, it is of paramount importance for most patients to be at home, reducing or postponing their attendance at clinics, as well as avoiding surgeries. In this scenario, neoadjuvant endocrine treatment could be an appropriate alternative treatment for hormone receptor positive breast cancer (luminal-like tumours) in order to minimise hospital admissions and to delay elective surgeries. Accordingly, we present a simple protocol that can be applied to most cases of luminal-like breast cancer and is appropriate for the majority of secondary or tertiary medical centres, or even primary care.

Since approximately 70%–75% of all breast cancers are oestrogen receptor (ER) positive, endocrine therapy constitutes one of the most important therapeutic approaches to these tumours. The inhibition of proliferative effect of oestrogens on cancer cells could be achieved either by reducing the oestrogen synthesis—or its active form— (as occurs with aromatase inhibitors or with LHRH agonists) or by blocking or modulating the ER (as happens with fulvestrant or tamoxifen) [1].

Conventionally, endocrine therapy in luminal tumours is conceived in the adjuvant setting, especially when treating localised disease, and its efficacy for preventing recurrences and increasing survival rates is well established [2, 3]. However, nowadays, the neoadjuvant approach has become one of the most useful strategies for treating some types of infiltrating breast cancer (Her2+, triple negative disease). It has many advantages: tumour and axillary downstaging—which allows for less extensive surgery, and therefore higher rates of breast conservation; assessment of in vivo response, permitting real-time evaluation of drug efficacy as well as the biological and molecular changes that can occur; and, finally, it provides an opportunity for testing new drugs, either individually or in combination. Assessing the response to neoadjuvant therapies also allows for a clinical prognosis to be established.

Nevertheless, while neoadjuvant chemotherapy is a common strategy, neoadjuvant endocrine treatments (NET) remain an underutilised tool that is quite often relegated to elderly or frail because of comorbidities non-chemotherapy candidates. Evidence for supporting this approach is quite strong. Robust (although relatively small) trial data show preoperative endocrine treatment to be a safe option when compared to chemotherapy [4–6], with significantly lower toxicity. The rates of breast conserving surgery increase clearly with NET, reaching 60%–80% [7–9]. It should be noted that pathological complete response rates are quite low [1] (as with chemotherapy), although this does not appear to influence prognosis as much as happens with non-luminal tumours. Even so, it is possible to establish a clinical prognosis based on the degree of the response. This was described by Ellis *et al* [10], after the results of the P024 study. They developed a prognostic model (Preoperative Endocrine Prognostic Index (PEPI) score) incorporating standard pathological staging variables and ‘on-treatment’ biomarker values, thereby establishing three risk groups [10]. Consequently, patients with a low PEPI score do not benefit from chemotherapy.

One of the main challenges with NET is to identify those tumours that will respond best to the treatment. It is well known that those cases with a strong ER expression (Allred scores 7–8) usually show more responsiveness. However, the intensity of ER positivity does not always reflect a good outcome to be derived from hormonal treatment. Other parameters, such as progesterone receptor expression, as well as changes produced in Ki67, also play a role in predicting responsiveness [11–14]. Performing biopsies a few weeks after starting the treatment provides information about possible resistance, especially in those cases where Ki67 is not reduced [13, 15–17]. Thus, NET offers an opportunity for investigating new biomarkers that may indicate a greater risk of resistance [18].

Finally, novel treatments that have been devised in the last few years can be combined with traditional endocrine drugs. They have demonstrated their efficacy in the metastatic/locally advanced setting (CDK4/6 inhibitors, PI3K or mTOR inhibitors, etc.) and are now providing promising data in the neoadjuvant scenario [19–21].

At our centre, we have been performing NET routinely for the past 3 years, and approximately 20% of our breast cancer patients are under such treatment. After the epidemiological emergency caused by COVID-19, we have simplified our own protocol and adapted it to the circumstances arising in our setting, so that it can be easily followed either at a breast unit or even at a primary-care centre.

In normal conditions, we consider NET for postmenopausal women with luminal-like/HER-2 negative breast cancers (ER+) [22] (2013—St Gallen criteria) larger than 1 cm. All cases are discussed in a multidisciplinary meeting. Aromatase inhibitors (AI) are the preferred drugs (letrozole is usually our first choice) although tamoxifen can be considered when AI intolerance exists. In cases where initial Ki67 is equal to or greater than 10%, we repeat a core biopsy after 4 weeks to check whether there is a reduction in this value and, therefore, we can assume this therapy to be efficacious. The follow-up is mostly carried out by the gynaecologist and, in some cases, by the medical oncologist. It usually consists of clinical exploration and ultrasound, following RECIST criteria, every 2 or 3 months. Exceptionally, MRI is employed, mostly in cases where an ultrasound follow-up is difficult or with some lobular breast cancers. If a reduction in tumour size is detected, treatment is maintained until maximum size reduction is achieved (usually in 6–12 months), after which surgery is generally performed. Radiotherapy is indicated, following conventional criteria, and chemotherapy remains an option if Ki67 is not reduced, a tumour progression is proven, or when there is a significant axillary involvement (Figure 1).

After the declaration of the epidemiological emergency and as soon as our hospital—like others around us—started to admit many COVID-19 patients, we were forced to reorganise all of the departments in order to free up rooms and mechanical ventilators. In addition, to protect people from contagion, patients are encouraged not to leave their homes. Consequently, priority is given to reducing or postponing attendance at the clinic as well as avoiding non-urgent surgeries.

In this new, abnormal situation, NET provides an opportunity for safely postponing breast cancer surgery, by using a systemic therapy that also avoids myelosuppression. As it is a simple treatment, it can even be explained to the patient, without any necessity for her to attend the clinic, either by the specialist or the primary care doctor, depending on each country’s circumstances.

We are suggesting a very simple protocol that is suitable for luminal-like infiltrating cancers and also for *in situ* ER+ disease. In our daily routine, NET is limited to postmenopausal patients, but, in the COVID-19 setting, we believe it can also be used with premenopausal patients, as it has also demonstrated its efficacy in this group [23].

- **Postmenopause:** Start treatment with any AI. Perform an ultrasound evaluation after 2 months. Continue treatment if the tumour size is stable or has reduced. If the tumour progresses, the therapeutic strategy should be changed (surgery/chemotherapy). In cases of AI intolerance, change to tamoxifen.
- **Premenopause:** Tamoxifen should be the first choice in low-risk cases. Ovarian function suppression (OFS) with goserelin combined with tamoxifen or AI could be considered in high-risk cases (positive nodes, very young age) [24], although it must be taken into account that OFS takes at least 15 days to be effective, so AI should not be incorporated during that first period. Follow up should be as explained above (Figure 2).

This strategy could be used for most cases of luminal-like breast cancer, even those with axillary positive nodes. A better response is expected when there is high ER expression (Allred score 6–8) or in luminal A phenotypes although our experience with over 150 patients shows that luminal B tumours respond almost as well.

Innate resistance to endocrine treatment might be expected in approximately 20%–30% of the cases [18]. In regular conditions, this can be suspected if Ki67 levels do not fall significantly after a few weeks of treatment; however, in this epidemiological emergency, repeating biopsies is probably unnecessary and ultrasound monitoring should provide a sufficient means for evaluating NET response. In any case, little harm is to be derived from treating a resistant tumour for 2 or 3 months and encountering such resistance can enable us to find a better approach later.

A conservative attitude in approach to luminal-like breast cancers in the presence of COVID-19 is also recommended by most scientific groups [25, 26] although it certainly must be adapted to suit each centre medical facility and the local conditions.

## Conclusion

In conclusion, in critical situations due to the pandemic, NET offers a safe and effective option for patients with luminal-like breast cancers and enables them to temporarily avoid surgery and visits to clinics.

## Conflicts of interest

The authors declare that they have no conflicts of interest.

## Funding statement

The authors have received no funds for the development of this work.

## Figures and Tables

**Figure 1.**
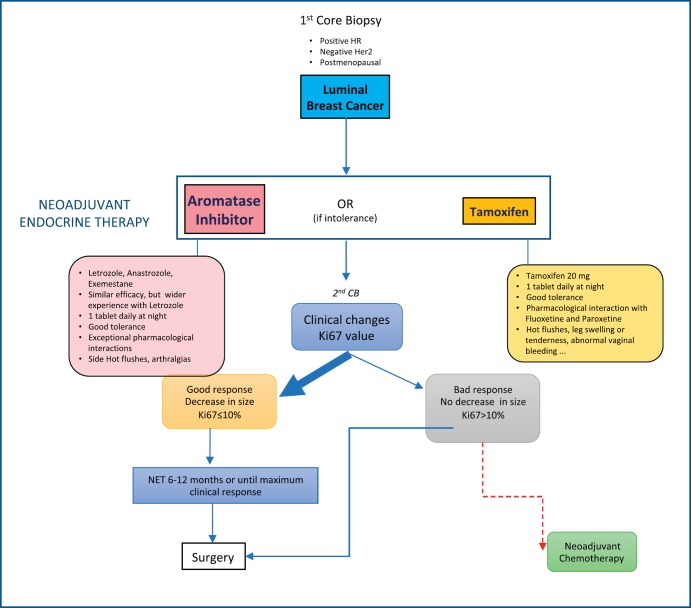
NET protocol in regular conditions.

**Figure 2.**
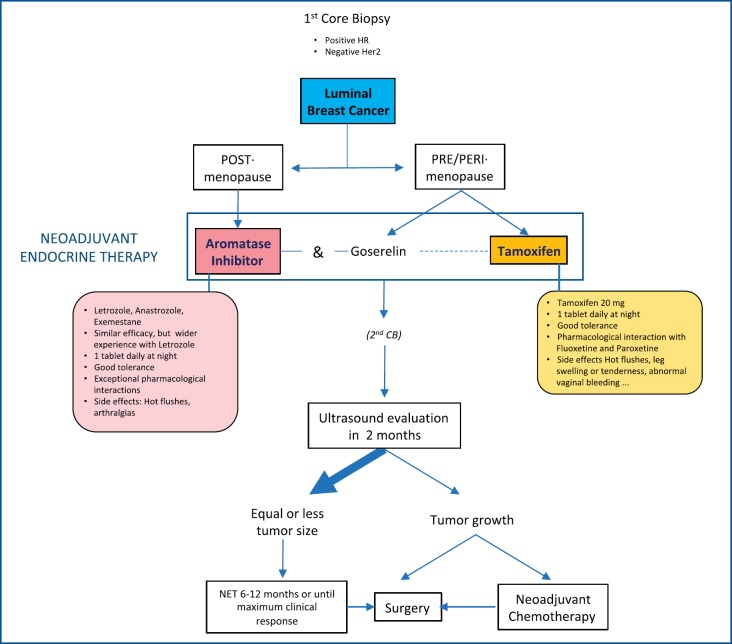
NET in COVID-19 scenario.﻿
